# Antecedent Dietary Glutamine Supplementation Benefits Modulation of Liver Pyroptosis in Mice with Polymicrobial Sepsis

**DOI:** 10.3390/nu12041086

**Published:** 2020-04-14

**Authors:** Man-Hui Pai, Jin-Ming Wu, Po-Jen Yang, Po-Chu Lee, Chun-Chieh Huang, Sung-Ling Yeh, Ming-Tsan Lin

**Affiliations:** 1 Department of Anatomy and Cell Biology, School of Medicine, College of Medicine, Taipei Medical University, Taipei 11031, Taiwan; pai0507@tmu.edu.tw; 2 Department of Surgery, National Taiwan University Hospital and College of Medicine, National Taiwan University, Taipei 10002, Taiwan; wujm0531@ntu.edu.tw (J.-M.W.); paulpjyang@gmail.com (P.-J.Y.); d97421103@ntu.edu.tw (P.-C.L.); yujiahcc@yahoo.com.tw (C.-C.H.); 3 School of Nutrition and Health Sciences, College of Nutrition, Taipei Medical University, Taipei 11031, Taiwan; sangling@tmu.edu.tw

**Keywords:** Sepsis, Glutamine, Liver pyroptosis, Caspase-1/11, Gasdermin D

## Abstract

The liver is the main organ responsible for bacterial and endotoxin clearance. Pyroptosis is a form of proinflammatory programmed cell death activated by caspase-1/11 and gasdermin D (GadD). Pyroptosis protects the host against bacterial infection; however, overactive pyroptosis can lead to organ injury. Glutamine (GLN) is a specific amino acid with anti-inflammatory and immunomodulatory properties. This study investigated the effects of GLN pretreatment on liver pyroptosis in a mouse model of polymicrobial sepsis. Mice were assigned to sham, sepsis control (Sepsis-C), and sepsis GLN (Sepsis-G) groups. The sham and Sepsis-C groups were fed the AIN-93G diet. The Sepsis-G group was provided with identical diet components except that part of the casein was replaced by GLN. After feeding the respective diets for 2 weeks, a cecal ligation and puncture (CLP) procedure was performed in the sepsis groups. An antibiotic was administered after CLP. Mice were sacrificed at either 24 or 72 h after CLP. The results showed that sepsis resulted in upregulated liver caspase-1/11 expression. Compared to the Sepsis-C group, the Sepsis-G group had higher liver caspase-11 and NLRP3 gene expressions at 24 h and lower active caspase-1/11 and cleaved GadD protein levels at 72 h after sepsis. Additionally, liver inflammatory cytokine gene expressions had decreased by 72 h post-CLP. The findings suggest that prophylactic administration of GLN initially upregulated liver pyroptosis to eradicate pathogens, yet the process of pyroptosis was suppressed in the late phase of sepsis. This may have beneficially attenuated liver inflammation and injury in an antibiotic-treated septic condition.

## 1. Introduction

Sepsis is a clinical syndrome of a systemic inflammatory response that commonly occurs in critically ill patients. It is defined as life-threatening multiorgan dysfunction triggered by a dysregulated immune response to infection [1]. In the United States, the incidence of sepsis is rising and the in-hospital mortality remains high at 25–30% [2]. The liver is the largest gland and a vital immune organ in the human body. This organ is responsible for various important physiological functions that makes it critical in the homeostasis of host metabolism and immunity [3,4]. The liver is one of the most frequently affected target organs in the clinical course of sepsis [5]. Sepsis is an uncontrolled immune response to pathogens [6]. As the first line of defense against microbial organisms in the blood, the liver is the major site of inflammatory responses encountering bacterial endotoxins during sepsis. Evidence has shown that sepsis-induced liver injury is an independent risk factor for multiple organ dysfunction [5,7]. Liver dysfunction and failure, especially those that occur as complications of severe sepsis, directly result in disease progression and death [8].

Programmed cell death is a fundamental process mediated by an intracellular program that is critical for tissue homeostasis and development in animals. However, dysregulation of this process is associated with the occurrence of a wide variety of diseases, including liver diseases [9]. Pyroptosis is a proinflammatory form of regulated cell death. Different from necrosis and apoptosis, pyroptosis is characterized by a caspase-1/4/5/11-dependent signaling pathway that initiates immune responses against intracellular bacteria [9,10]. There are two different pyroptosis pathways. Canonical pyroptosis begins with nod-like receptor family pyrin domain containing 3 (NLRP3) inflammasome signaling and subsequent caspase-1 activation [10,11]. Pathogen-associated molecule patterns (PAMPs) recognized by the NLRP3 inflammasome initiate canonical pyroptosis [12]. Non-canonical pyroptosis is mediated by caspase-11 (caspase-4/5 in humans), which can be directly activated by lipopolysaccharide (LPS) [13]. Activation of caspase-1/11 leads to the cleavage of the pyroptotic substrate, gasdermin D (GadD), and subsequent interleukin (IL)-1β and IL-18 secretion, which promotes proinflammatory cytokine production [9,14]. However, excessive host cell pyroptosis may induce an inflammatory cascade and ultimately cause tissue damage and diseases [14]. Various cells in the liver, including macrophages, stellates, and hepatocytes, participate in pyroptosis, which can directly and indirectly cause liver cell injury [15,16,17].

Glutamine (GLN), an abundant free amino acid in the body, was found to have anti-inflammatory and immune-regulatory properties in catabolic and stressed conditions [18,19]. Numerous studies demonstrated that GLN supplementation had favorable effects on mitigating sepsis-induced organ injury including of the lungs, kidneys, intestines, etc. by various mechanisms [20,21,22,23,24]. However, no study has investigated the influences of GLN on liver pyroptosis during sepsis. Since GLN was found to reduce proinflammatory cytokine production and decrease mortality in a lipopolysaccharide-treated animal model [25], we hypothesized that GLN supplementation may modulate liver pyroptosis and alleviate liver injury induced by sepsis. This study used cecal ligation and puncture (CLP) to induce sepsis, because it is a well-established murine model to mimic the progression and characteristics of polymicrobial sepsis in humans [26]. In order to mimic a critical care setting, saline and an antibiotic were administered after CLP.

## 2. Materials and Methods

### 2.1. Animals

Male C57BL/6 mice at the age of 5 weeks were obtained from the National Laboratory Animal Center (NLAC; Taipei, Taiwan). All mice were raised in a humidity (50–55%) and temperature (21 ± 2 °C) controlled room with a 12 h light/dark cycle and acclimation for 1 week. Three mice were housed in one cage. A common semi-purified diet formulated by the American Institute of Nutrition (AIN-93G) were fed ad libitum during the acclimation period. The protocol of the study was approved by the Institutional Animal Care and Use Committee of Taipei Medical University (LAC-2018-0040). The care of the mice was in compliance with the latest Guide for the Care and Use of Laboratory Animals (National Research Council, 2011).

### 2.2. Study Procedures

Mice weighing 22–25 g were randomly grouped into a sham control (sham, *n* = 12) group and two sepsis groups. Mice in the sham group and sepsis control group (Sepsis-C, *n* = 20) were provided an AIN-93G diet. The sepsis GLN group (Sepsis-G, *n* = 20) was given a GLN-enriched diet based on AIN-93G diet except that GLN replaced part of the casein. GLN provided 25% of the total amino acid nitrogen in this diet. This dosage of GLN used was reported to have anti-inflammation and immune-regulatory properties in rodents [23,24]. The semi-purified diet and the GLN-enriched diets were similar in energy and macronutrient distribution. The two diets were isonitrogenous (Table 1). The respective diets were fed to the mice for 2 weeks; then, the CLP procedure was carried out in the two sepsis groups. A laparotomy operation with cecum exposed but without ligation and puncture was performed in the sham group. The operation of CLP is described briefly as follows. Mice were anesthetized with an intraperitoneal injection of zoletil (25 mg/kg body weight (BW); Virbac, Carros, France) and Rumpon (10 mg/kg BW; Bayer, Leverkusen, Germany). The abdominal wall was incised about 1 cm to open the peritoneum. The cecum was exposed and was ligated with 3-0 silk at approximately 50% below the ileocecal valve. A 22-gauge needle was used to puncture the distal cecum in two places. A small amount of feces was extruded into the peritoneal cavity; then, the cecum was placed back to the abdomen. The abdominal wound was closed with a continuous suturing technique. Before skin closure, 100 μL of 0.25% bupivacaine was provided at the incision site to relieve pain. An antibiotic, Ertapenem, was injected (75 mg/kg BW) at 6 h and mice were sacrificed at either 24 or 72 h after CLP. BWs were recorded daily during the experimental period. All mice were anesthetized and then euthanized by cardiac puncture. Blood samples were collected in tubes containing heparin. The whole blood was centrifuged at 700× *g* and 4 °C for 15 min to obtain plasma. The liver was harvested for further analysis.

### 2.3. Measurements of Plasma Biochemical Parameters

Liver function markers, including aspartate aminotransferase (AST) and alanine aminotransferase (ALT), were measured using commercial kits (BioVision, Milpitas, CA, USA). Concentrations of interleukin (IL)-1β and IL-18 in plasma samples were quantified by enzyme-linked immunosorbent assay (ELISA) kits (R&D Systems, Minneapolis, MN, USA). Procedures followed the manufacturer’s instructions.

### 2.4. RNA Extraction and Quantitative Reverse-Transcription Polymerase Chain Reaction (RT-qPCR) of Liver Tissues

Total RNA in liver tissues was isolated using Trizol reagent (Invitrogen, Waltham, MA, USA). Then, up to 2.5 μg of total RNA was used for reverse-transcription by a RevertAid First Strand cDNA Synthesis Kit (Thermo Scientific, Waltham, MA, USA) with oligo (dT)18 primers. A real-time PCR was performed in the Rotor-Gene Q 5plex HRM System (Qiagen, Venlo, Netherlands). Primers used in this study are listed in Supplementary Table S1. The expression of each gene was assayed in a total volume of 25 μL containing 1× Maxima SYBR Green/ROX qPCR Master Mix (Thermo Scientific), 200 nM of each primer, and 50 ng of complementary (c)DNA. PCR thermal cycling conditions for cDNA amplification were 50 °C for 2 min, 95 °C for 10 min, and 40 cycles of 95 °C for 15 s and 60 °C for 1 min, followed by a final dissociation curve analysis that was used to confirm the specificity of the real-time PCR. The temperature of the melt curve ranged from 60 °C to 95 °C. Multiples of change of messenger (m)RNA were calculated by the equation 2^−ΔΔCt^ (ΔCt is the difference of threshold cycles between the target gene and internal control (glyceraldehyde-3-phosphate dehydrogenase, GAPDH), and ΔΔCt is the difference in ΔCt between the sepsis and sham groups) [27]. We duplicated the measurement of each sample and the number of the sample in each group is at least six. There is no significantly different in the expression (Ct value) of GAPDH in all groups [28].

### 2.5. Western Blotting Analysis for Protein Expressions in the Liver

Total proteins were extracted from the liver using radioimmunoprecipitation assay (RIPA) buffer (Sigma-Aldrich, St. Louis, MO, USA) with a protease inhibitor (Sigma-Aldrich) and phosphatase inhibitor (Roche, Basel, Switzerland) cocktail. Protein concentrations were determined using the Bradford assay reagent (Bio-Rad, Hercules, CA, USA). To prepare loading samples, 20 μg of protein was mixed with Laemmli sample buffer (Sigma-Aldrich). Protein samples were separated by sodium dodecylsulfate polyacrylamide gel electrophoresis (SDS-PAGE) and transferred onto a polyvinylidene difluoride membrane. After being blocked with BlockPRO™ Blocking Buffer (Visual Protein, Taipei, Taiwan), membranes were incubated with anti-gasdermin D (GadD; 1:1000; Cell Signaling Technology, Danvers, MA, USA), anti-caspase-1 (1:1000; Abcam, Cambridge, UK), anti-caspase-11 (1:1000; Sigma-Aldrich), or anti-β-actin antibodies (1:5000; Sigma-Aldrich) in Tris-buffered saline with Tween 20 (TBST) containing 1% bovine serum albumin overnight at 4 °C. Then, membranes were washed three times for 10 min each with TBST and revealed by goat anti-rabbit, goat anti-rat, or goat anti-mouse horseradish peroxidase-conjugated secondary antibodies (Jackson ImmunoResearch, West Grove, PA, USA) for 1 h at room temperature, followed by an enhanced chemiluminescence reaction. Using the BioSpectrum^®^ Imaging System (UVP, Upland, CA, USA), specific bands were visualized and photographed.

### 2.6. Histopathology of the Liver

Tissues were first embedded in paraffin and were sectioned at 5 μm. The specimens were mounted on glass slides, and stained with hematoxylin and eosin (H&E). An Olympus BX43 light microscope (Tokyo, Japan) equipped with a digital camera (Canon, Tokyo, Japan) was used to take the digital images at 100× magnification per section. To determine cytoplasmic swelling in liver tissues, five fields per section were examined. Cytoplasmic swelling was examined by hepatocellular ballooning and scored by a pathologist who was blinded to the experimental groups. The hepatocyte ballooning analysis was performed by semiquantitative scoring according to Kleiner et al. [29] as follows: 0, none; 1, a few ballooned cells; 2, many ballooned cells/prominent ballooning.

### 2.7. Statistical Analysis

Data are expressed as the mean ± standard error of the mean (SEM). All statistical analyses were performed using GraphPad Prism 5.0 software [30]. To analyze the differences among groups, a one-way analysis of variance (ANOVA) with the Bonferroni post-hoc test was used. Values were considered statistically significant at *p* < 0.05.

## 3. Results

There were no differences in the initial BWs among the three groups. The sepsis groups had lower BWs than the sham group at 24 h (Sham 27.2 ± 0.3 g vs. Sepsis-C 26.1 ± 0.3 g and Sepsis-G 26.5 ± 0.3 g, *p* < 0.05), and more weight loss was observed at 72 h after CLP (Sham 27.3 ± 0.5 g vs. Sepsis-C 24.5 ± 0.5 g and Sepsis-G 24.9 ± 0.4 g, *p* < 0.05). No differences in BWs were seen between the Sepsis-C and Sepsis-G groups at either 24 or 72 h after CLP. All mice in Sham group and sepsis groups at 24 h post-CLP survived. Some animals died at 72 h after CLP; however, there was no difference in mortality rates between the two sepsis groups (Sepsis-C 25% vs. Sepsis-G 23%, *p* > 0.05).

### 3.1. Plasma Biochemical Markers and Inflammatory Cytokine Concentrations

In both sepsis groups at 24 h after CLP, plasma levels of AST and IL-18 had increased two-fold compared to the sham group. The Sepsis-G group even had higher ALT and IL-1β concentrations than the sham and Sepsis-C groups. By 72 h, AST levels in the Sepsis-C group were still significantly higher than those of the sham and Sepsis-G groups. However, there were no differences in AST, ALT, IL-1β, or IL-18 levels between the sham and Sepsis-G groups. (Table 2).

### 3.2. Pyroptosis-Related Gene Expressions in the Liver after CLP

**Figure 1:**
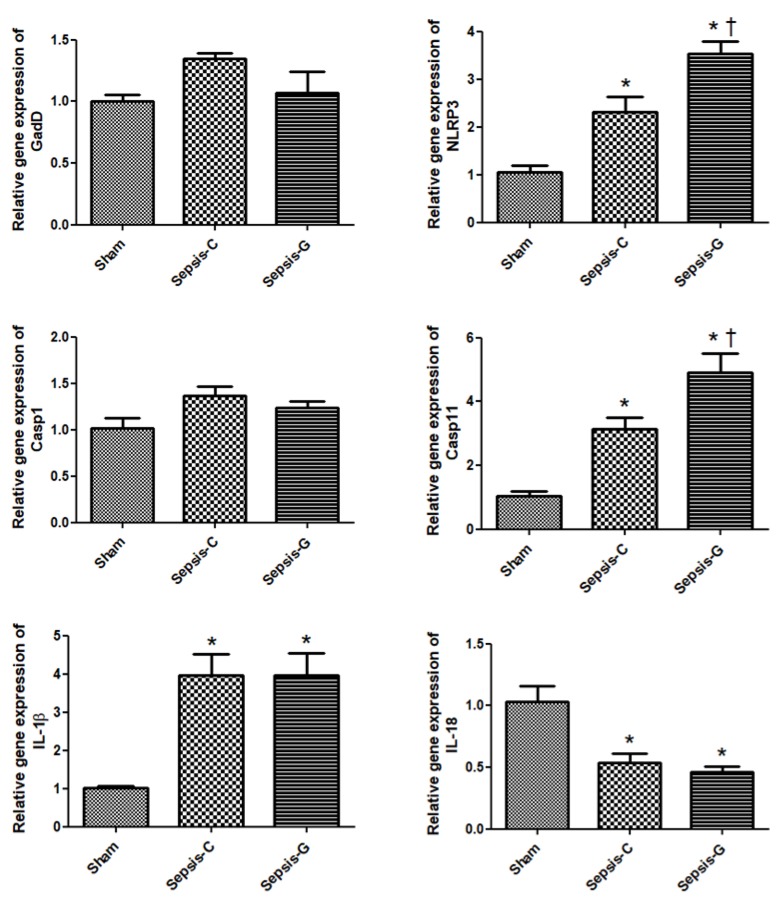
Expressions of genes related to pyroptosis in the liver 24 h after cecal ligation and puncture (CLP). Sham: control group with a sham operation; Sepsis-C: control group with CLP; Sepsis-G: glutamine (GLN) group with a CLP operation. Quantitation of mRNA changes was analyzed by a real-time Polymerase Chain Reaction (PCR) and was calculated by the comparative cycle threshold CT (2^−ΔΔCt^) method. mRNA expression levels in the sham control group were used as a calibrator. Data are shown as the mean ± standard error of the mean (SEM). *n* = 8 for each group. Differences among the sham, Sepsis-C, and Sepsis-G groups were analyzed by a one-way analysis of variance (ANOVA) with the Bonferroni post-hoc test. * Significantly differs from the sham group. + Significantly differs from the Sepsis-C group (*p* < 0.05).

**Figure 2:**
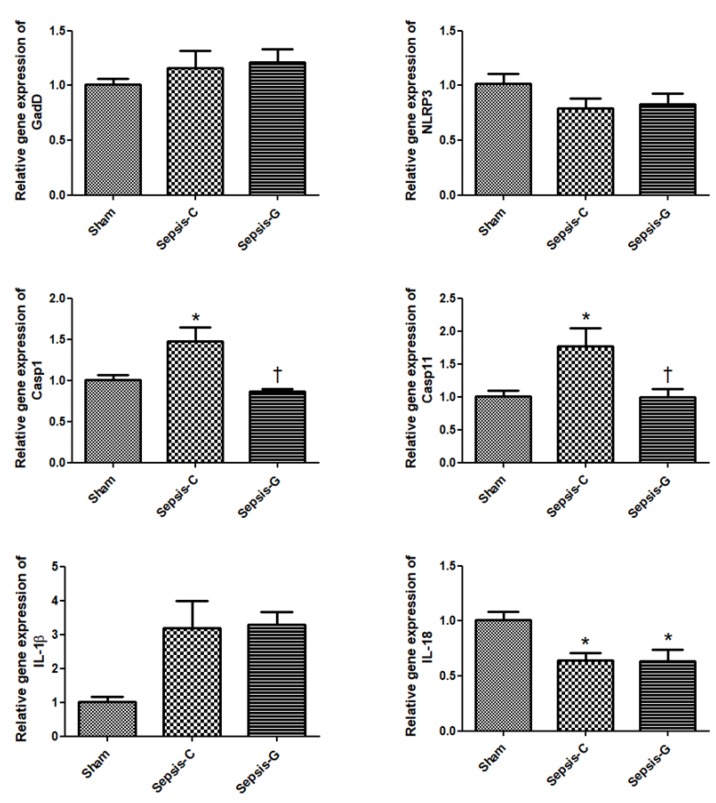
Expressions of genes related to pyroptosis in the liver 72 h after cecal ligation and puncture (CLP). Sham: control group with a sham operation; Sepsis-C: control group with CLP; Sepsis-G: glutamine (GLN) group with a CLP operation. Quantitation of mRNA changes was analyzed by a real-time PCR and was calculated by the comparative CT (2^−ΔΔCt^) method. mRNA expression levels in the sham control group were used as a calibrator. Data are shown as the mean ± SEM. *n* = 8 for each group. Differences among the sham, Sepsis-C, and Sepsis-G groups were analyzed by a one-way ANOVA with the Bonferroni post-hoc test. * Significantly differs from the sham group. + Significantly differs from the Sepsis-C group (*p* < 0.05).

Both sepsis groups had higher gene expressions of NLRP3, caspase-11, and IL-1β and lower expression of IL-18 compared to the sham group. The Sepsis-G group had higher caspase-11 and NLRP3 expressions than the Sepsis-C group by 24 h post-CLP (Figure 1 and Figure 2). When comparing the sepsis groups at 72 h, the Sepsis-G group had lower expression levels of caspase-1 and caspase-11. There were no differences in caspase-1 and caspase-11 gene expressions between the sham and Sepsis-G groups at 72 h post-CLP (Figure 2).

### 3.3. Inflammatory-Related Gene Expressions in the Liver after CLP

**Figure 3:**
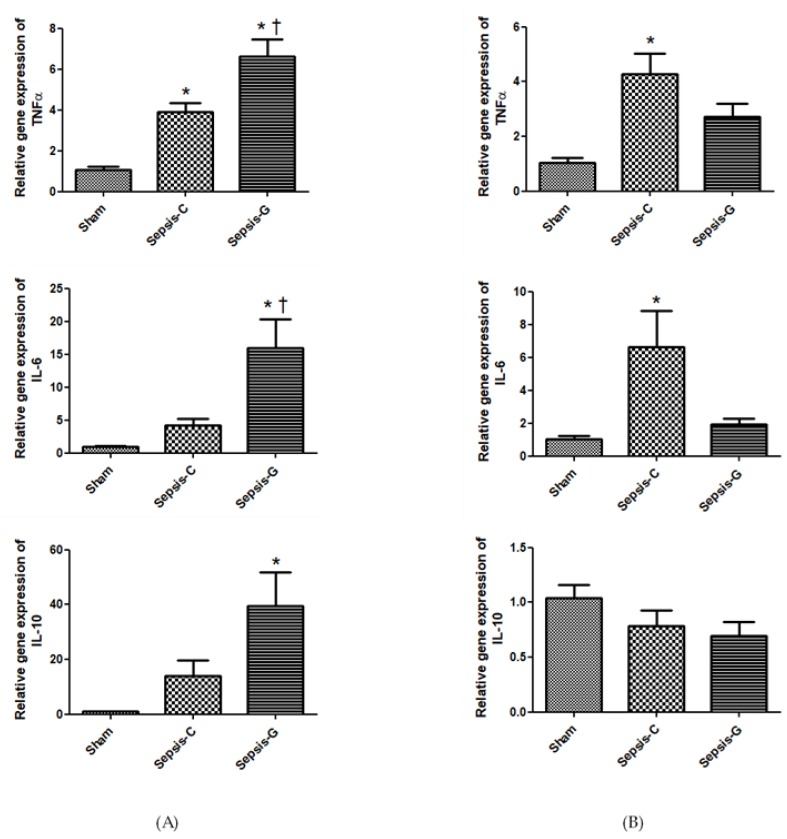
Expressions of tumor necrosis factor (TNF)-α, interleukin (IL)-6 and IL-10 genes in the liver (**A**) 24 and (**B**) 72 h after cecal ligation and puncture (CLP). Sham: control group with a sham operation; Sepsis-C: control group with CLP; Sepsis-G: glutamine (GLN) group with a CLP operation. Quantitation of mRNA changes was analyzed by a real-time PCR and was calculated by the comparative CT (2^−ΔΔCt^) method. mRNA expression levels in the sham control group were used as a calibrator. Data are shown as the mean ± SEM. *n* = 8 for each group. Differences among the sham, Sepsis-C, and Sepsis-G groups were analyzed by a one-way ANOVA with the Bonferroni post-hoc test. * Significantly differs from the sham group. + Significantly differs from the Sepsis-C group (*p* < 0.05).

By 24 h after the operation, the Sepsis-G group exhibited higher tumor necrosis factor (TNF)-α, IL-6, and IL-10 gene expressions than the sham group and higher TNF-α and IL-6 expressions than those of the Sepsis-C group. When comparing the Sepsis-G group to the sham and Sepsis-C groups at 72 h, only the Sepsis-C group demonstrated significant increases in TNF-α and IL-6 expressions. There were no differences in TNF-α or IL-6 between the Sepsis-G and sham groups (Figure 3).

### 3.4. Protein Expression Levels of Caspase-1/11 and GadD in the Liver at 72 h after CLP

**Figure 4:**
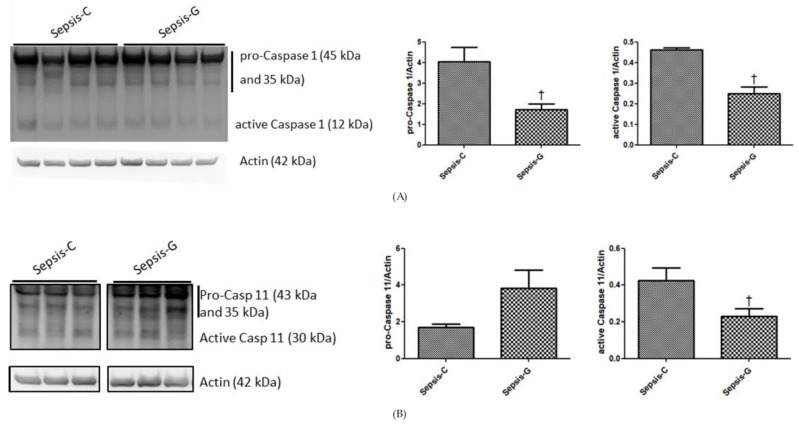

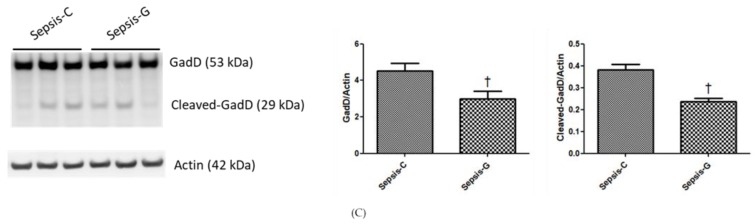
Protein levels of (**A**) pro- and active caspase-1, (**B**) pro- and active caspase-11, and (**C**) gasdermin D (GadD) and cleaved GadD in liver tissues. Whole-tissue lysates were analyzed by immunoblotting, and β-actin was used as a loading control. Densitometric analysis of the blot corrected by the protein loading control. Sham: control group with sham operation; Sepsis-C: control group with cecal ligation and puncture (CLP); Sepsis-G: glutamine (GLN) group with a CLP operation. Results of the densitometric analysis are shown as the mean ± standard error of the mean (SEM). Differences among the sham, Sepsis-C, and Sepsis-G groups were analyzed by a one-way ANOVA with the Bonferroni post-hoc test. ^†^ Significantly differs from the Sepsis-C group (*p* < 0.05).

The Sepsis-G group showed lower total GadD (53 kDa), cleaved GadD (29 kDa), active caspase-11 (30 kDa), and pro- (45 and 35 kDa) and active caspase-1 (12 kDa) protein expression levels than those in the Sepsis-C group (Figure 4A–C).

### 3.5. Histopathological Aspects of the Liver

**Figure 5:**
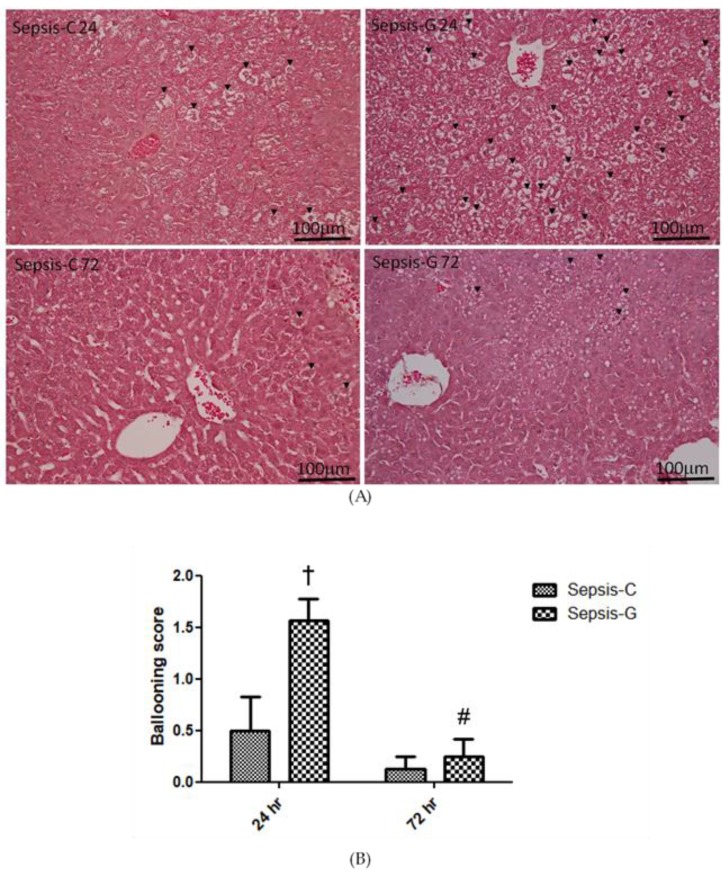
Histopathology of liver tissues. (**A**) Hematoxylin and eosin staining. Representative histological images are shown at 100× magnification. Cellular ballooning was indicated by arrows. (**B**) hepatocellular ballooning scores of the liver. Sham: control group with a sham operation; Sepsis-C: control group with cecal ligation and puncture (CLP); Sepsis-G: glutamine (GLN) group with a CLP operation. Data are presented as the mean ± standard error of the mean (SEM). ^†^ Significantly differs from the Sepsis-C group at 24 h after CLP. ^#^ Significantly differs from the Sepsis-G group at 24 h after CLP *(p* < 0.05).

Hepatocellular ballooning was more severe and the swelling score was higher in the Sepsis-G group than the Sepsis-C group at 24 h after the operation, whereas this phenomenon had improved by 72 h post-CLP. The extents of cellular ballooning were less and the scores were lower at 72 h after CLP in both sepsis groups (Figure 5A,B).

## 4. Discussion

In this study, all septic mice treated with an antibiotic survived at 24 h and 75–77% survived at 72 h post-CLP. The survival rate was much higher than of mice without an antibiotic, which reached only 50% survival by 24 h after CLP (unpublished data). Antibiotic administration is part of the standard protocol for treating critical patients with polymicrobial infection. The experimental design of our study is clinically relevant for investigating sepsis-induced remote organ injury. A previous study reported that CLP-induced acute liver injury and pyroptosis were distinct at 24 h postoperatively [11]. Antibiotic treatment post-CLP may delay the progression of sepsis-associated liver pyroptosis observed in this study. The findings of this study showed that pretreatment with GLN increased liver caspase-11 and NLRP3 gene expressions at 24 h, while caspase-1/11 and GadD protein levels were downregulated at 72 h after sepsis. These results indicated that liver pyroptosis is initially upregulated yet decreases in the late phase of sepsis, and administration of GLN may attenuate liver dysfunction and injury.

During bacterial infection, as in sepsis, the liver is the main organ responsible for bacterial and toxin clearance [31,32]. Several studies showed that hepatic pyroptosis plays an important role in various infectious and non-infectious diseases [33,34,35]. Caspase-1/11-mediated pyroptosis stimulates immune responses that recruit neutrophils and phagocytes to the site of infection and help eradicate pathogens. Pyroptosis is considered an effective host defense mechanism against bacterial infections [10]. However, excessive pyroptosis may cause inflammation and organ injury. A previous study found that the pyroptosis rate is positively correlated with liver injury [11]. In this study, we found that caspase-1 and caspase-11 gene expressions in the liver post-CLP were both upregulated, indicating that pyroptosis occurred after sepsis. Because cytoplasmic swelling is one of the features of pyroptosis, the difference in hepatocellular ballooning between the two sepsis groups was evaluated by H&E staining and a scoring system in this study.

The findings of this study demonstrated that the sepsis group with GLN administration exhibited increased gene expressions of caspase-11 and NLRP3 in the liver at 24 h post-CLP. NLRP3 is a member of the nucleotide-binding oligomerization domain (NOD)-like receptor family that is essential for activating caspase-1 [10]. In contrast to expression levels observed at 24 h, caspase-1/11 genes accompanied by active caspase-1 and caspase-11 and cleaved GadD protein levels were downregulated by 72 h. GadD is a shared component of caspase-1/11-mediated pyroptosis and a critical factor in the antibacterial response [36]. Activation of caspase-1 and caspase-11 leads to the proteolytic cleavage of GadD that initiates the process of pyroptosis and subsequent inflammatory responses [10]. Since caspase-1/11 activation initiates pyroptosis, which promotes the recruitment of phagocytes, pretreating mice with GLN may enhance bacterial clearance at an earlier phase of sepsis (24 h post-CLP). Although inflammatory cytokines (IL-6 and TNF-α) were enhanced in response to upregulated pyroptosis, anti-inflammatory cytokines (IL-10) were also produced to balance the inflammatory response at this time point. Lower levels of IL-18 found in both sepsis groups can be explained by the overexpression of IL-10, because there is an inverse regulatory function between IL-10 and IL-18 [37,38].

**Figure 6:**
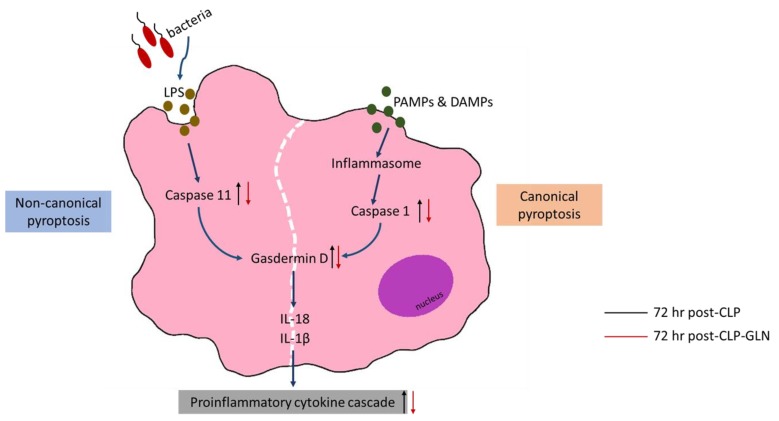
A schematic diagram illustrating the effects of dietary glutamine supplementation on liver pyroptosis in mice with polymicrobial sepsis 72 h after cecal ligation and puncture. PAMPs, pathogen-associated molecule patterns; DAMP, damage-associated molecule patterns; LPS, lipopolysaccharide; IL-18, interleukin 18; IL-1β, interleukin-1β.

The downregulated expressions of caspase-1/11 and GadD in the late phase (72 h post-CLP) in the GLN sepsis group cannot be explained by immunosuppression. ALT and AST are normally presented in hepatocytes. With cellular injury, these enzymes leak into circulation and are considered indicators of liver injury [3]. We observed that plasma ALT and AST levels were reduced and IL-10 remained comparable to the sham control, indicating that the immune response was not suppressed and liver function had improved by this time point. Uncontrolled pyroptosis may become detrimental to sepsis [10]. A study performed by Kayagaki et al. [36] found that mice lacking GadD were protected from a lethal dose of LPS, an endotoxin found in outer membrane of gram-negative bacteria. Additionally, Chen et al. [11] reported that inhibition of hepatocyte pyroptosis prevented sepsis-induced liver injury. Suppression of overactive caspase-1/11- and GadD-mediated pyroptosis in the late phase of sepsis as observed in the GLN-supplemented group in our study may have benefits of attenuating liver injury (the schematic diagram is shown in Figure 6). The histological findings of hepatocyte ballooning observed in this study were consistent with liver pyroptosis-associated gene and protein expressions, in that more cytoplasmic swelling occurred initially but had subsequently improved by 72 h post-CLP.

Sepsis is a syndrome with systemic inflammation and high oxidative stress. A previous multicenter clinical trial performed by Heyland et al. [39] revealed that GLN administration increased mortality in critically ill septic patients. Since organ dysfunction may impair the ability to use GLN in the body, the outcomes mentioned above might not apply to all catabolic conditions. Despite disease severity, GLN dosage used and timing of administration may also critical for the outcomes. A clinical trial proved that GLN supplementation was safe in intensive care unit patients [40]. Additionally, a systematic review concluded that parenteral GLN administered after resolution of shock and multiorgan failure reduces both the hospital stay and mortality [41]. A previous study revealed that GLN administration attenuated the release of inflammatory cytokines (TNF-α, IL-6, and IL-18) in septic mice [42]. An in vitro study found that GLN reduced IL-1β-mediated inflammatory cytokine production in human intestinal mucosa [43]. On the other hand, reactive oxygen species are stimuli of inflammasome activation, and reducing oxidative stress may block inflammasome-mediated signal pathways [44]. GLN is the precursor of glutathione, which is an important antioxidant for providing tissue protection against oxidative stress [45]. Studies performed by Tsai et al. [46] also showed that GLN supplementation decreased oxidative gene expressions and increased the antioxidant capacity in diabetic rats. The anti-inflammatory property and GLN-associated redox-based reactions may be partly responsible for suppressing hepatic pyroptosis in sepsis.

There were limitations in this study. Cell pyroptosis has several specific characteristics such as pores in plasma membranes, cell swelling, and osmotic lysis. Cytoplasmic swelling is a feature shared with apoptotic cells [10]. The histological findings observed in this study could not differentiate between apoptosis and pyroptosis. This may explain why there were no differences in cellular ballooning scores between the two sepsis groups at 72 h after CLP. Electron microscopy or combined staining with both Annexin V and propidium iodide could be used to differentiate different cell death modes [10]. In addition, parameters of oxidative stress in the liver were not measured in this study. The distinct features of pyroptosis and the possible mechanisms responsible for reducing pyroptosis require further investigation.

## 5. Conclusions

This is the first study to investigate the influence of dietary GLN supplementation on hepatocyte pyroptosis during sepsis. The findings showed that sepsis resulted in liver pyroptosis and inflammation. Prophylactic GLN supplementation with the dosage used for 2 weeks in this study upregulated capase-1 and caspase-11 mediated liver pyroptosis at an early phase, while it downregulated pyroptosis in the late phase of sepsis and improved liver function. These new findings provide basic information that GLN may modulate the balance of liver pyroptosis at different sepsis stages, which may have benefits in attenuating liver inflammation and injury in an antibiotic-treated septic condition. The results of this study provide basic information and imply that antecedent GLN use may be organ-protective for patients with at high risk for sepsis, such as after major operations, of old age, and with multiple comorbidities. 

## Figures and Tables

**Table 1:** Composition of the experimental diets (g/kg).

Components	Control Diet	Glutamine Diet
Soybean oil	70	70
Casein	200	150
Glutamine	0	41.7
Sucrose	100	100
Mineral mixture ^†^	35	35
Vitamin mixture ^‡^	10	10
Fiber	50	50
Choline bitartrate	2.5	2.5
L-Cysteine	3	3
Corn starch	529.5	537.8

^†^ The ingredients of the mineral mixture are as following (mg/g): calcium phosphate dibasic, 500; sodium chloride, 74; potassium sulfate, 52; potassium citrate monohydrate, 20; magnesium oxide, 24; manganese carbonate, 3.5; ferric citrate, 6; zinc carbonate, 1.6; cupric carbonate, 0.3; potassium iodate, 0.01; sodium selenite, 0.01; and chromium potassium sulfate, 0.55. ^‡^ The ingredients of vitamin mixture are as following (mg/g): thiamin hydrochloride, 0.6; riboflavin, 0.6; pyridoxine hydrochloride, 0.7; nicotinic acid, 3; calcium pantothenate, 1.6; D-biotin, 0.05; cyanocobalamin, 0.001; retinyl palmitate, 1.6; DL-α-tocopherol acetate, 20; cholecalciferol, 0.25; and menaquinone, 0.005.

**Table 2:** Plasma liver function markers and interleukin (IL)-1β and IL-18 levels among groups at different time points.

	24 h Post-CLP			72 h Post-CLP		
	Sham	Sepsis-C	Sepsis-G	Sham	Sepsis-C	Sepsis-G
ALT (U/L)	6.04 ± 2.15	10.08 ± 1.08	25.18 ± 8.13 *	1.47 ± 0.20	9.20 ± 3.80	2.48 ± 0.42
AST (U/L)	31.45 ± 9.15	72.67 ± 4.12 *	72.33 ± 11.23 *	17.38 ± 4.82	49.69 ± 10.66 *	25.96 ± 2.70
IL-1β (pg/mL)	5.10 ± 0.75	7.77 ± 1.58	12.53 ± 1.86 *	7.18 ± 1.27	10.67 ± 1.35	8.92 ± 1.55
IL-18 (pg/mL)	14.42 ± 3.83	46.70 ± 6.71 *	38.87 ± 8.04 *	23.97 ± 6.38	20.91 ± 3.58	24.60 ± 4.56

CLP, cecal ligation and puncture; ALT, alanine aminotransferase; AST, aspartate aminotransferase; Sepsis-C: control group with CLP; Sepsis-G: glutamine (GLN) group with CLP. * Significantly differs from the sham group.

## Supplementary Materials

The following are available online at https://www.mdpi.com/2072-6643/12/4/1086/s1, Table S1: Oligonucleotide sequence of primers for the PCR.
