# Supplementary material for: Antecedent Dietary Glutamine Supplementation Benefits Modulation of Liver Pyroptosis in Mice with Polymicrobial Sepsis
Source: Nutrients. 2020 Apr 14;12(4):1086. doi: 10.3390/nu12041086 (PMC7230693; doi:10.3390/nu12041086)
Supplement: Supplementary file 1 [file nutrients-12-01086-s001.pdf]

Supplemental Table 1

Oligonucleotide sequence of primers for the PCR.

Target	Direction	Oligonucleotide sequence (5’-3’)	GenBank accession no.
GadD	forward	CCAGTGCCTCCATGAATGTGT	NM_026960.4
	reverse	TCACCACAAACAGGTCATCCC	
Casp 1	forward	AATGAAGTTGCTGCTGGAGGA	NM_009807.2
	reverse	CAGAAGTCTTGTGCTCTGGGC	
Casp 11	forward	TCATTTTACTCTGTCAAGCTGTCT	NM_007609.3
	reverse	GTAAGGACTTCTTTGCCCAGC	
NLRP3	forward	AGAGCCTACAGTTGGGTGAAATG	NM_145827.4
	reverse	CCACGCCTACCAGGAAATCTC	
IL-1β	forward	TGCCACCTTTTGACAGTGATG	NM_008361.4
	reverse	ATGTGCTGCTGCGAGATTTG	
IL-18	forward	CAGGCCTGACATCTTCTGCAA	NM_008360.2
	reverse	TCTGACATGGCAGCCATTGT	
TNFα	forward	GCCTCTTCTCATTCCTGCTTG	NM_013693.3
	reverse	CTGATGAGAGGGAGGCCATT	
IL-6	forward	TACCACTTCACAAGTCGGAGGC	NM_031168.2
	reverse	CTGCAAGTGCATCATCGTTGTTC	
IL-10	forward	AAGGCAGTGGAGCAGGTGAA	NM_010548.2
	reverse	CCAGCAGACTCAATACACAC	
GAPDH	forward	TGTGTCCGTCGTGGATCTGA	NM_008084.3
	reverse	TTGCTGTTGAAGTCGCAGGAG	

GadD, gasdermin D; Casp1, caspase1; Casp 11, caspase 11; NLRP3, nod-like receptor family pyrin domain containing 3; TNF-α, tumor necrosis factor alpha; IL-1β, interleukin-1β; IL-18, interleukin 18; IL-6, interleukin-6; IL-10, interleukin-10; GAPDH, glyceraldehyde 3-phosphate dehydrogenase

Formatted: Font color: Auto
